# Disparities in Secondhand Smoke Exposure in the United States: National Health and Nutrition Examination Survey 2011-2018

**DOI:** 10.1001/jamainternmed.2020.3975

**Published:** 2020-11-23

**Authors:** Surendra S. Shastri, Rajesh Talluri, Sanjay Shete

**Affiliations:** 1 Department of Health Disparities Research, The University of Texas MD Anderson Cancer Center, Houston; 2 Department of Data Science, The University of Mississippi Medical Center, Jackson; 3 Department of Epidemiology, The University of Texas MD Anderson Cancer Center, Houston; 4 Department of Biostatistics, The University of Texas MD Anderson Cancer Center, Houston; 5 Division of Cancer Prevention and Population Science, The University of Texas MD Anderson Cancer Center, Houston

## Abstract

This cross-sectional study examines National Health and Nutrition Examination Survey data to assess the prevalence of secondhand smoke exposure in nonsmokers in the US population 3 years and older.

Secondhand smoke exposure (SHSe) is one of the causes of sudden infant death syndrome, respiratory tract infections, ear infections, and asthma attacks in infants and children; coronary heart disease, stroke, and lung cancer in adult nonsmokers; and low birth weight, premature deliveries, and congenital defects in pregnancies.^1^ It results in nearly 42 000 deaths (more than 41 000 adults and 900 infants) among nonsmokers every year in the US, with Black individuals accounting for 24% to 36% of the infant deaths.^2^ The US Surgeon General determined that there is no risk-free level of SHSe.^3^ With the outbreak of coronavirus disease 2019, which affects lung function, improving smoke-free policies to enhance air quality should be a growing priority.

## Methods

This study was conducted using data from 4 cycles of the National Health and Nutrition Examination Survey (NHANES) from 2011 to 2018. The most recent data (2017-2018) were released in March 2020. The NHANES is designed to monitor nationwide health and uses a complex, multistage probability design to sample the noninstitutionalized civilian population residing in all 50 states.^4^ Sensitive and personal questions are administered through computer-assisted personal interview and the audio computer-assisted self-interview. The biological specimens for laboratory testing were collected at a mobile examination center. Among nonsmokers, individuals having serum cotinine levels of 0.05 to 10 ng/mL were considered to have SHSe. The primary outcome of this study is the prevalence of SHSe in nonsmokers in the US population 3 years and older. Because the NHANES data are deidentified and publicly available, this secondary data analysis was exempt from institutional review board approval and informed consent in accordance with the Common Rule and University of Texas MD Anderson Cancer Center policy.

The survey-adjusted weights were used to estimate the prevalence of SHSe in nonsmokers. A survey logistic regression was used to test for trends in SHSe over the 4 two-year intervals (2011-2012, 2013-2014, 2015-2016, and 2017-2018) of NHANES. Also, a multivariable survey logistic regression was performed to identify factors associated with SHSe. For all statistical analyses, *P* values were calculated using Wald test statistic, and significance was defined as 2-sided *P* value ≤ .05. The analysis was performed using the survey package in R, version 4.0.0 (R Foundation for Statistical Computing).

## Results

This study found that disparities in SHSe among non-Hispanic Black individuals compared with people of other races and among those below the poverty levels have persisted throughout 2011 to 2018 (Table 1). For example, in the 2017-2018 cycle, the SHSe prevalence continued to be twice as high among nonsmoker non-Hispanic Black individuals (48.02%) compared with non-Hispanic White individuals (22.03%) and among those living below the poverty level (44.68%) compared with those living above the poverty level (21.33%). Similarly, children aged 3 to 11 years continued to experience SHSe at high rates (38.23%). Multivariable logistic regression identified younger age (odds ratio [OR], 1.88, for 12-19 years, and OR, 2.29, for 3-11 years), non-Hispanic Black race/ethnicity (OR, 2.75), less than high school education (OR, 1.59), and living below the poverty level (OR, 2.61) as risk factors for SHSe in the 2017 to 2018 cycle, with little change across all data cycles (Table 2).

**Table 1.** Survey-Weighted Percentage of Nonsmokers 3 Years and Older With Serum Cotinine Levels 0.05 to 10 ng/mL, by Selected Sociodemographic Characteristics—National Health and Nutrition Examination Survey, 2011-2018

Variable	Secondhand smoke exposure, % (95% CI)				*P* value^a^
	2011-2012	2013-2014	2015-2016	2017-2018	
Overall	25.31 (22.61-28.21)	25.34 (21.09-30.13)	24.55 (20.92-28.58)	24.65 (21.53-28.06)	.68
Sex					
Male	27.69 (24.83-30.73)	27.16 (22.87-31.91)	24.28 (19.78-29.42)	26.59 (21.89-31.90)	.50
Female	23.34 (20.50-26.44)	23.79 (19.06-29.27)	24.78 (21.51-28.36)	23.02 (20.53-25.73)	.99
Age					
3-11 y	40.59 (34.17-47.36)	38.02 (31.08-45.49)	38.16 (33.37-43.19)	38.23 (34.23-42.39)	.56
12-19 y	33.80 (28.45-39.61)	32.18 (25.13-40.13)	32.45 (27.76-37.52)	33.22 (28.14-38.72)	.90
≥20 y	21.30 (18.76-24.09)	22.10 (18.30-26.42)	21.09 (17.58-25.09)	21.18 (17.97-24.79)	.83
Race/ethnicity					
Mexican American	23.85 (17.10-32.23)	19.91 (15.90-24.64)	20.60 (16.50-25.41)	16.61 (12.24-22.14)	.10
Other Hispanic	22.30 (15.10-31.64)	25.00 (19.13-31.96)	21.60 (17.98-25.71)	21.20 (17.11-25.96)	.62
Non-Hispanic					
White	21.76 (18.76-25.10)	21.65 (16.25-28.23)	22.31 (17.49-28.00)	22.03 (18.46-26.07)	.86
Black	46.82 (38.21-55.61)	50.18 (44.46-55.90)	45.72 (39.68-51.89)	48.02 (41.25-54.87)	.97
Education					
Less than high school	28.46 (23.91-33.50)	32.67 (27.68-38.09)	26.48 (19.20-35.30)	27.52 (22.04-33.77)	.46
High school or above	20.18 (17.39-23.29)	20.51 (16.66-24.97)	20.31 (16.80-24.33)	20.46 (17.14-24.23)	.93
Poverty level					
Above	21.08 (18.78-23.58)	21.28 (17.29-25.91)	21.43 (17.58-25.85)	21.33 (18.76-24.15)	.88
Below	43.25 (37.44-49.25)	47.09 (40.77-53.50)	41.17 (33.45-49.36)	44.68 (38.25-51.29)	.91
Housing					
Owned or being bought	19.03 (16.24-22.17)	19.21 (14.84-24.50)	19.17 (15.40-23.60)	Data not released	.95
Rented	36.82 (32.45-41.41)	38.59 (33.73-43.69)	35.64 (29.35-42.46)	Data not released	.76

^a^ *P* values are based on the 2-sided test for assessing trends in secondhand smoke exposure over time.

**Table 2.** Multivariable Logistic Regression Results Showing Adjusted Odds Ratios (ORs) of Nonsmokers Having Serum Cotinine Levels 0.05 to 10 ng/mL—National Health and Nutrition Examination Survey, 2011-2018

Variable	2011-2012		2013-2014		2015-2016		2017-2018	
	OR (95% CI)	*P* value	OR (95% CI)	*P* value	OR (95% CI)	*P* value	OR (95% CI)	*P* value^a^
Sex								
Male	1.37 (1.22-1.54)	.001	1.30 (1.11-1.51)	.02	0.94 (0.79-1.12)	.52	1.22 (0.96-1.55)	.15
Female	1 [Reference]							
Age								
3-11 y	2.32 (1.76-3.05)	.001	1.84 (1.52-2.23)	.002	2.22 (1.84-2.69)	<.001	2.29 (1.81-2.90)	<.001
12-19 y	1.77 (1.37-2.30)	.004	1.68 (1.33-2.13)	.007	1.88 (1.48-2.38)	.004	1.88 (1.43-2.48)	.004
≥20 y	1 [Reference]							
Race/ethnicity								
Mexican American	0.6 (0.40-0.91)	.046	0.49 (0.31-0.76)	.03	0.52 (0.38-0.71)	.01	0.48 (0.33-0.68)	.006
Other Hispanic	0.57 (0.40-0.81)	.02	0.71 (0.42-1.19)	.25	0.57 (0.40-0.82)	.03	0.73 (0.48-1.10)	.18
Non-Hispanic								
White	1 [Reference]							
Black	2.23 (1.64-3.01)	.001	2.62 (1.87-3.66)	.002	2.08 (1.51-2.86)	.007	2.75 (2.12-3.58)	<.001
Education								
Less than high school	1.57 (1.16-2.13)	.02	1.69 (1.28-2.24)	.01	1.36 (0.93-2.00)	.18	1.59 (1.19-2.12)	.02
High school or above	1 [Reference]							
Poverty level								
Above	1 [Reference]							
Below	2.06 (1.74-2.43)	<.001	2.50 (2.04-3.07)	<.001	1.97 (1.57-2.47)	.002	2.61 (2.04-3.35)	<.001
Housing								
Owned or being bought	1 [Reference]						Data not released	
Rented	2.03 (1.62-2.55)	<.001	1.85 (1.48-2.30)	.003	1.98 (1.51-2.59)	.004	Data not released	

^a^ *P* values presented are based on the 2-sided Wald test.

## Discussion

Although the prevalence of SHSe among nonsmokers in the US declined substantially (87.5% to 25.3%) from 1988 to 2012,^5^ progress has stagnated since then, with persisting racial and economic disparities. Populations with a lower socioeconomic status have higher smoking rates, lower knowledge about health risks of tobacco, higher risk of workplace exposure, and higher likelihood of living in low-income multi-unit housing and have their communities targeted more by tobacco companies, which would possibly explain the high SHSe observed in our study. Furthermore, in households with smokers, non-Hispanic Black individuals are less likely to have a complete smoking ban in homes, while parents of any race or ethnicity with a child on Medicaid or uninsured (a proxy for lower income) are less likely to have a complete smoking ban in family vehicles. We conclude that more needs to be done to implement enhanced and equitable comprehensive smoke-free laws throughout the US (currently implemented in only 27 states). The serum cotinine levels in nonsmokers provide a measure of overall SHSe, regardless of the sources or locations of exposure; therefore, these laws should be expanded to include other forms of vaping and should include private properties (eg, cars) for meaningful reduction in SHSe among vulnerable populations (eg, young children). The primary limitation of the study is the potential nonresponse bias in the NHANES data collection, although our study is survey weighted to account for the nonresponse.
